# Spontaneous Pneumomediastinum from Running Sprints

**DOI:** 10.1155/2010/927467

**Published:** 2010-09-02

**Authors:** Joseph W. Turban

**Affiliations:** Department of Surgery, The University of Hawaii John A. Burns School of Medicine, 651 Ilalo Street, 3rd Floor, MEB Honolulu, HI 96813, USA

## Abstract

Spontaneous pneumomediastinum (SPM) is a fairly rare condition, caused by increased intrathoracic pressure, leading to free air in the mediastinal structures. Underlying lung conditions are associated with increased incidence of SPM, including asthma, interstitial lung disease, pneumonia, bullous lung, and radiation therapy for lung cancer. It is often preceded by Valsalva maneuvers, vomiting, coughing, asthma exacerbation, sneezing, childbirth, or intense physical activity. A case of SPM is presented in a 15-year-old male, who complained of throat pain and dyspnea while running sprints at football practice. Workup revealed SPM, and he was subsequently admitted and treated conservatively. His symptoms resolved in 2 days and he was discharged and suffered no further recurrences. In contrast to secondary pneumomediastinum, SPM is usually a benign condition although life-threatening conditions can rarely arise. Differentiating between these two conditions has important prognostic indications. There is a paucity of prospectively collected data regarding SPM, and considerable variation in recommendations concerning the extent of workup.

## 1. Introduction

Spontaneous pneumomediastinum (SPM)—pneumomediastinum without an obvious precipitating cause—is a fairly rare condition, caused by increased intrathoracic pressure, leading to free air in the mediastinal structures. Although SPM is generally considered a benign condition, secondary pneumomediastinum may be due to a serious medical condition, such as perforation of an internal organ seen in Boerhaave syndrome.

This is a case of a 15-year-old male who suffered from SPM on the first day of American football practice while running sprints in a conditioning exercise. The etiology of SPM is discussed along with the common signs and symptoms associated with this condition.

## 2. Case Presentation

A fifteen-year-old male presented at 0100 to the local emergency department complaining of increasing dyspnea, chest pain and a sore throat. He denied any nasal discharge, cough, nausea or vomiting. His symptoms had started the previous afternoon on the first day of football practice. He had been running 24 100-yard sprints as a conditioning exercise, and about 2/3-way through, he started becoming symptomatic. There had been no contact drills. He had been able to complete the exercise, but his symptoms continued to worsen throughout the evening. He never had a similar episode. He did not recall any Valsalva-like maneuvers, nor did he lift any weights. 

He denied being a smoker and had no past history of asthma, respiratory problems, or other significant health issues. He denied any illicit or recreational drug use.

On exam, the patient was a 6′3′′, 270 lbs, large teenager who appeared slightly dyspneic, but not distressed. Blood pressure was 128/70, heart rate 80, respiratory rate 28, and temperature 97.8 F; SaO_2_ was 97% on room air. HEENT exam was unremarkable, and the oral cavity showed normal appearing tonsils and a midline uvula. Speech was normal sounding, with no nasal quality. There was no erythema and no exudates. There was no subcutaneous emphysema or crepitus in the neck or the chest area. Lungs were clear to auscultation bilaterally with no adventitial lung sounds. CV showed a regular rate and rhythm with no murmurs, clicks, gallops, or extra heart sounds, specifically no systolic crepitations. Abdomen was benign, and the rest of the exam was unremarkable.

**Figure 1:**
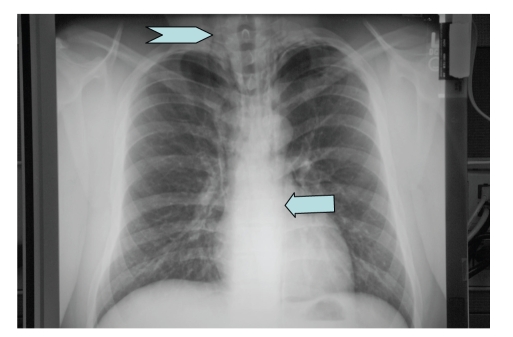
Initial chest X-ray showed subcutaneous emphysema, with air tracking into the neck area bilaterally (chevron), and a para-aortic air stripe of the left (arrow).

Chest X-ray (Figure 1) showed subcutaneous emphysema, with air tracking into the neck area bilaterally, and a para-aortic air stripe of the left. There was also evidence of a minimal left apical pneumothorax (PNX). The trachea was midline, and the remainder of the lung fields and cardiac silhouette were normal.

**Figure 2:**
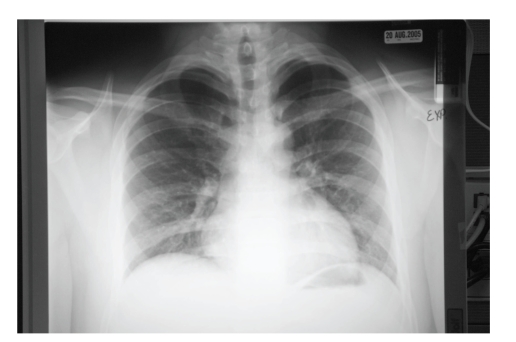
Follow-up chest X-ray, expiratory view, two weeks later, shows resolution of previous findings.

The patient was diagnosed with pneumomediastinum and transferred to a tertiary care facility where he was admitted, placed on high flow oxygen, and observed. Later that morning, he was still noted to be dyspneic and complaining of chest pain. The following morning, the patient was asymptomatic, and was discharged. At a followup visit to his pediatrician 2 weeks later, the chest X-ray (Figure 2, expiration view) showed complete resolution of his previous lung findings, and he was given clearance to return to physical activity.

## 3. Discussion

Pneumomediastinum, or mediastinal emphysema, is the presence of free air in the mediastinal structures. It is usually credited as first being described in 1819, by the pathologist R. T. Läennec, who first observed this condition in trauma patients [1].

Primary or Spontaneous Pneumomediastinum (SPM) is caused by increased pressure gradient between the alveoli and pulmonary interstitium [2]. Secondary causes include gas-forming microorganisms during an infection, and increasing intrathoracic pressure from trauma causing a disruption of bronchiolar, alveolar, or rarely, esophageal tissue, with leakage of air into the interstitial tissues [3, 4].

Spontaneous Pneumomediastinum has no apparent secondary cause, and is relatively rare in adults, (1 out of 44,511 ED visits) [5]. Children have increased frequency (1 in 800 to 1 in 15,150 ED visits) [6]. The initial description and defining of this condition is credited to Hamman in 1939 [7] although reports of spontaneous pneumomediastinum date back to the 17th century, where it was observed in extreme effort during childbirth [2].

 The mechanism in SPM, as outlined by Macklin [2], is disruption of the alveolar tissues (the Macklin phenomenon [4]). Increased intra-alveolar pressure causes air to leak into the interstitium and via centripetal forces (the Macklin effect [7]) to collect centrally [5]. This process is driven by pressure differences between the mediastinum and the lung parenchyma during the respiratory cycle [4]. Common causes of increased intra-alveolar pressure include acute asthma exacerbation, Valsalva maneuvers, childbirth, intense athletic activity, coughing or vomiting. Decrease in intrapleural pressure or in the lung interstitium, as in vasoconstriction, or loss of integrity of the alveolar-capillary membrane, as in interstitial lung disease, predisposes patients to SPM. Inhalation drugs, such as cocaine, may precipitate SPM by multiple mechanisms of vasoconstriction and interstitial lung disease [4, 7, 8].

Most sports-related cases of pneumomediastinum involve contact although SPM has been reported after scuba diving and synchronized swimming [9].

Pneumomediastinum differs from pneumothorax in that, in the latter, there is a disruption of the parietal pleura with collection of air in the pleural space; in pure pneumomediastinum the pleura remains intact.

The epidemiology of SPM varies depending on the study. Gender distribution has been reported as equal [10] although many series show a predominance in males (57–87%) [4, 7, 8, 10–12]. It is most common in the 2nd and 3rd decades of life [7].

Underlying lung conditions are associated with increased incidence of SPM. Asthma (8–39%), interstitial lung disease (18%), pneumonia, bullous lung, and radiation therapy for lung cancer have all been associated with SPM [5, 7, 12].

Frequent preceding events include vomiting (24–36%), asthma exacerbation (15–24%), coughing (7–35%), sneezing (13%), breath holding, physical activity (30%), increased effort during labor (4–15%), and screaming and even snorting, a sharp exhale as a sign of psychological stress [13]. One series identified a 55% rate of cocaine consumption in the hours prior to presentation [8]. Anorexia nervosa has also been associated with SPM; it has been suggested a decrease in pulmonary tissue elasticity found in this condition may predispose patients to alveolar air leakage [14]. Up to 32–66% of incidences of SPM have no identified precipitating event [4, 7, 10–12].

Symptoms include chest pain (26–70%), dyspnea (26–46%), cough (26–45%), sore throat (18%), neck pain (4–38%), rhinolalia (65%), hoarse voice (65%), neck and/or chest swelling (87%) [7, 8, 10, 12]. 

Signs include systolic crepitations (Hamman's sign), an abnormal crunchy or bubbling sound heard during cardiac auscultation (8–17%), subcutaneous emphysema (12–100%), neck swelling (14%), and hoarse voice and rhinolalia (65%) [1, 4, 6–8, 10–12].

In an exclusive series of children (9 months to 18 years), Lee found a similar distribution of symptoms, signs, and preceding events and PNX (17%) [6].

Diagnosis is made most commonly by chest X-ray, which reveals subcutaneous emphysema, and pneumomediastinum. Small pneumomediastinum not seen on chest X-ray will be seen on chest CT although the use of CT only to confirm the diagnosis of SPM in an otherwise well-appearing patient is probably not warranted [11]. Ruling out other causes of pneumomediastinum is important, as secondary pneumomediastinum can be associated with a poor outcome [10].

Treatment is most often conservative; complications, although not common, include tension pneumopericardium; massive subcutaneous emphysema causing clinical effects can be drained with a silastic drain [7]. Concurrent pneumothorax is found in 6–32% of cases in SPM [8, 10, 12], and should be treated conventionally. Of the 20 out of 62 (32%) patients reported by Iyer to have a concurrent PNX, only 6 had tube thoracostomy. The 13 patients with PNX admitted without chest tube placement had the same length of stay as those without PNX [12]. Rarely, tension pneumomediastinum occurs, requiring surgical drainage and placement of a drain. In the absence of gastrointestinal complaints or findings, further study of the esophagus and GI tract is not recommended [11]. The use of antibiotics in the absence of obvious infection is also not recommended [4].

Resolution of free air typically takes 2–4 days; recurrence is rare, but has been reported [10], often related to inhalant recreational drug use [12]. Patients with underlying medical conditions, such as bullous lung disease, interstitial lung disease, or graft versus host disease have a more prolonged hospital course. In these cases, the hospital course is often determined more by an underlying condition rather than SPM.

A review of the literature reveals most articles regarding SPM are retrospective case series; as such, their information is dependent on relative completeness of documentation. Patients may have had more symptomatology then the treating physicians reported in the patient's chart. There was also significant variability in their patient populations, and in the inclusion criteria as to what constituted SPM. Considerable disparity in recommendations exists between authors, with no clear agreement on the use of CT, hospitalization, or workup for potential esophageal disruption. Also problematic is the diagnosis of SPM cannot be made until other causes of pneumomediastinum have been ruled out. The distinction between SPM and secondary pneumomediastinum is important; causes of secondary pneumomediastinum, such as Boerhaave syndrome, can carry high mortality and morbidity rates. This may necessitate an extensive workup, including further diagnostic studies. Some authors recommend chest CT to all patients to rule out undiagnosed/unrecognized chest pathology [8].

This is a case of a 15-year-old male who presented with SPM following vigorous noncontact exercise. He had no identifiable underlying lung conditions. The presumed pathophysiology in this case appears to be increased intrathoracic pressure occurring during extreme physical exercise. Although the patient denied recalling any Valsalva-like maneuvers, the effect of running multiple sprints could certainly raise intrathoracic pressure. The association between extreme exertion and SPM is will documented [4, 7, 8, 10–12]. In the absence of GI symptoms, no further esophageal or GI workup was performed. No chest CT was performed. The patient was observed, and was asymptomatic 36 hours later and was discharged. Repeated CXR was normal after 2 weeks. There is limited prospectively acquired data regarding workup and management of SPM. In the era of cost containment, a prospective study looking at optimizing medical resources for SPM may be warranted. GI workup may be reserved for patients with significant GI symptoms [11]. Small concomitant PNX can be managed conservatively. Most patients are asymptomatic 24–48 hours after presentations. IV antibiotics do not appear necessary in the absence of obvious infections.
